# Coping and Caregiver Burden and Depression Among Chinese Caregivers of Older Adults With Cognitive Impairment

**DOI:** 10.1093/geroni/igab046.942

**Published:** 2021-12-17

**Authors:** Kaipeng Wang, Xiang Gao

**Affiliations:** 1 University of Denver, Denver, Colorado, United States; 2 Huazhong University of Science and Technology, Wuhan, Hubei, China (People's Republic)

## Abstract

Coping strategies are important factors that influence caregivers’ mental health outcomes. The purpose of this study is to examine the association between coping strategies and caregiver burden and depression among Chinese caregivers of older adults with cognitive impairment. Data came from structured interviews with 300 primary family caregiver-care recipient dyads in Wuhan, China. We used OLS to examine the association between coping strategies and caregiver burden and depression. More positive reframing and acceptance were associated with lower caregiver burden, whereas more self-distraction was associated with higher caregiver burden. More positive reframing was associated with lower caregiver depression, whereas higher self-distraction and religion were associated with higher caregiver depression. Findings of this study suggest that a psychosocial intervention package that emphasizes on enhancing positive reframing skills and affirming acceptance may be effective in reducing caregiver burden and depression among Chinese caregivers of older adults with cognitive impairment.

